# Online Provision of *BRCA1* and *BRCA2* Health Information: A Search Engine Driven Systematic Web-Based Analysis

**DOI:** 10.3390/cancers16132324

**Published:** 2024-06-25

**Authors:** Tamar A. Gootzen, Ashwin Kalra, Katrina Sarig, Monika Sobočan, Samuel George Oxley, Nina Dworschak, Ariadni Georgiannakis, Sevasti Glynou, Angeliki Taniskidi, Subhasheenee Ganesan, Michelle Ferris, Rosa Legood, Ros Eeles, D. Gareth R Evans, Caitlin T. Fierheller, Ranjit Manchanda

**Affiliations:** 1 Centre for Cancer Screening, Prevention & Early Diagnosis, Wolfson Institute of Population Health, Charterhouse Square, Queen Mary University of London, London EC1M 6BQ, UK; t.gootzen@qmul.ac.uk (T.A.G.); a.kalra@qmul.ac.uk (A.K.); k.sarig@qmul.ac.uk (K.S.); monika.sobocan3@um.si (M.S.); s.oxley@qmul.ac.uk (S.G.O.); s.ganesan@qmul.ac.uk (S.G.); c.fierheller@qmul.ac.uk (C.T.F.); 2 Department of Gynaecological Oncology, Royal London Hospital, Barts Health NHS Trust, London E1 1BB, UK; 3 Department of Obstetrics and Gynecology, Faculty of Medicine, University of Maribor, Taborksa ul, 2000 Maribor, Slovenia; 4 Barts and the London School of Medicine, Queen Mary University of London, London E1 2AD, UK; n.k.dworschak@smd19.qmul.ac.uk (N.D.); a.georgiannakis@smd20.qmul.ac.uk (A.G.); s.glynou@smd20.qmul.ac.uk (S.G.); a.taniskidi@smd20.qmul.ac.uk (A.T.); 5 Lane End Medical Practice, London HA8 9GJ, UK; michelle.ferris@nhs.net; 6 Department of Health Services Research and Policy, London School of Hygiene and Tropical Medicine, London WC1H 9SH, UK; rosa.legood@lshtm.ac.uk; 7 The Institute of Cancer Research, and Royal Marsden NHS Foundation Trust, London SM2 5PT, UK; ros.eeles@icr.ac.uk; 8 Manchester Centre for Genomic Medicine, Division of Evolution, Infection and Genomic Sciences, University of Manchester, MAHSC, 6th Floor Saint Mary’s Hospital, Manchester M13 9WL, UK; gareth.d.evans@manchester.ac.uk; 9 MRC Clinical Trials Unit at UCL, Institute of Clinical Trials and Methodology, Faculty of Population Health Sciences, University College London, London WC1V 6LJ, UK

**Keywords:** genetic testing, *BRCA*, online information, Jewish

## Abstract

**Simple Summary:**

*BRCA* genetic testing is available for UK Jewish individuals through the National Health Service or private providers. This study evaluated how well UK organisations (UKO), UK Jewish community organisations (JCO), and genetic testing providers (GTP) provide information about *BRCA* online. Google was used to find relevant websites and assessed the first 100 links. We reviewed the sites for how accessible, comprehensive, detailed, accurate, and high-quality the information was, giving each site a score out of 5. From 6856 search results, we found 45 UKOs, 16 JCOs, and 18 GTPs that provided *BRCA* information. While most sites (84%) were easy to access, the information was often incomplete. Only 35% of sites covered more than half of the important *BRCA* topics. Most sites mentioned breast and ovarian cancer (82%), but fewer mentioned other *BRCA*-related cancers. Overall, the quality of information was low-to-moderate. This highlights a need for better online *BRCA* information.

**Abstract:**

*BRCA* genetic testing is available for UK Jewish individuals but the provision of information online for *BRCA* is unknown. We aimed to evaluate online provision of *BRCA* information by UK organisations (UKO), UK Jewish community organisations (JCO), and genetic testing providers (GTP). Google searches for organisations offering *BRCA* information were performed using relevant sets of keywords. The first 100 website links were categorised into UKOs/JCOs/GTPs; additional JCOs were supplemented through community experts. Websites were reviewed using customised questionnaires for *BRCA* information. Information provision was assessed for five domains: accessibility, scope, depth, accuracy, and quality. These domains were combined to provide a composite score (maximum score = 5). Results were screened (*n* = 6856) and 45 UKOs, 16 JCOs, and 18 GTPs provided *BRCA* information. Accessibility was high (84%,66/79). Scope was lacking with 35% (28/79) addressing >50% items. Most (82%, 65/79) described *BRCA*-associated cancers: breast and/or ovarian cancer was mentioned by 78%(62/79), but only 34% (27/79) mentioned ≥1 pancreatic, prostate, melanoma. Few websites provided carrier frequencies in the general (24%,19/79) and Jewish populations (20%,16/79). Only 15% (12/79) had quality information with some/minimal shortcomings. Overall information provision was low-to-moderate: median scores UKO = 2.1 (IQR = 1), JCO = 1.6 (IQR = 0.9), and GTP = 2.3 (IQR = 1) (maximum-score = 5). There is a scarcity of high-quality *BRCA* information online. These findings have implications for UK Jewish *BRCA* programmes and those considering *BRCA* testing.

## 1. Introduction

*BRCA1* and *BRCA2* pathogenic variants (PVs) are associated with significantly increased lifetime risks of cancers of the breast, ovary, pancreas, prostate, and melanoma [1,2,3]. A number of effective risk management options are available for breast cancer (BC) and ovarian cancer (OC) prevention in *BRCA* PV carriers, including screening (e.g., mammography/MRI for BC), preventive medication (e.g., tamoxifen or anastrozole for BC), risk-reducing surgery (e.g., risk-reducing mastectomy (RRM) for BC or risk-reducing salpingo-oophorectomy (RRSO) for OC prevention) [4,5,6], and pre-implantation genetic diagnosis [7,8,9,10,11].

Public awareness, availability, and accessibility of *BRCA* genetic testing has increased over time. Traditionally, genetic testing provision has been restricted to individuals meeting strict clinical/family history criteria with a pre-test *BRCA* probability of ≥10% [12]. Genetic testing is available free of cost through the UK National Health Service (NHS) or via private testing providers at a charge. Implementation of mainstream genetic testing for cancer diagnosis and associated cascade testing is identifying more unaffected *BRCA* carriers. A London pilot UK NHS programme of *BRCA* testing for all women diagnosed with BC commenced in July 2023. Long-term trials have demonstrated feasibility, acceptability, high satisfaction, reduced anxiety, no detrimental impact on quality of life, and cost-effectiveness of population-based *BRCA* testing in the Jewish population [13,14,15,16]. Resultantly, NHS England Cancer Programme introduced clinical implementation of population-based *BRCA1* and *BRCA2* testing for all UK Jewish adults in January 2024 [17,18,19]. Consequently, there is an even stronger demand and need for *BRCA* information for individuals considering testing and to support identified PV carriers [20]. Although clinicians/health professionals (including clinical geneticists, genetic counsellors, medical/surgical oncologists, clinical nurses, and others) provide pre- and post-test counselling, many individuals require and seek further information and support from online resources. According to the UK Office of National Statistics, 60% of UK adults searched for health-related information online in 2020 [21]. This highlights the need for high-quality *BRCA* information online as the demand for testing increases and information is sought online.

There has been very limited research into the availability and quality of online information on *BRCA.* One study, not specific to *BRCA* information, of UK direct-to-consumer genetic testing services found very limited compliance with good practice principles developed by the UK Human Genetics Commission [22]. There has been no research into the quality and provision of *BRCA* specific information provided by community organisations, charities, cancer organisations, or testing providers.

Given the interest in, need for, and impact of online information on user behaviour and health-related decisions, it is crucial for healthcare organisations, advocacy/patient support/charity groups, and other stakeholders to offer accurate and easily accessible information about *BRCA1*/*BRCA2*. Several websites provide online information, support, and other services relating to *BRCA*; these include cancer organisations and community-based organisations with interests in health and community support. Organisations working with the UK Jewish community may also provide information about *BRCA* given the higher carrier frequency of *BRCA* PVs in the Jewish population (1:40 compared to ~1:200 for the general UK population). Furthermore, several companies offer private genetic testing, either via clinician or direct-to-consumer, with associated online resources and testing information. These may be promoted with search engine advertisements or optimisations and are likely to be read by interested individuals.

This study aimed to evaluate the online provision and quality of *BRCA1*/*BRCA2* information, including that related to genetic testing and its implications, by UK organisations (UKO), UK Jewish community organisations (JCO), and (private) genetic testing providers (GTP).

## 2. Materials and Methods

### 2.1. Organisation Groups

UKOs were defined as UK-based organisations offering information regarding *BRCA*-associated cancers (breast, ovarian, pancreatic, prostate cancer, or melanoma), *BRCA* testing, or *BRCA* support. JCOs were defined as organisations working primarily in the UK Jewish community in areas of health, welfare, community education/awareness raising, signposting, or community leadership. GTPs were defined as private companies offering *BRCA1* and *BRCA2* testing to UK residents.

### 2.2. Search Strategy

One author (A.K.) performed online searches (www.google.co.uk, accessed on 27 February 2022) between February to May 2022, using Mozilla Firefox version-97.0.1 (64-bit), from a UK desktop PC, with pre-defined sets of keywords relating to *BRCA* information to identify websites (Supplementary File: A). To avoid bias from personalised search algorithms, as described previously [23], we used a non-university/NHS internet connection, a private browser window, and changed IP-address and deleted browser data/history before each search.

Forty searches were performed to identify UKOs and JCOs websites with *BRCA* information. For every search with different keywords, details, and website links of the first 100 search results (including advertisements) were recorded. GTP websites were identified through 40 keyword searches and supplemented by the UKO/JCO search results. Website links were recorded for the first 100 (UKO and JCO) and 50 GTP search results, as results thereafter only included duplicates. Additional JCO websites were identified through lists from the Jewish Leadership Council, Board of Directors, Jewish Charity Guide, and three Jewish community experts. Duplicates were removed.

### 2.3. Website Selection

We included organisations based on their mission/aims/remit, which on assessment should provide/support information relevant to *BRCA*. This included UKOs focused on *BRCA*-associated cancers, *BRCA* genetic testing, *BRCA* support, or cancer in general. We included JCOs with stated interests of Jewish community health, providing health-related emotional or wellbeing support and/or health education or focused on women’s health or cancer.

We included UKO or JCO websites from organisations with a national reach that provided *BRCA* information in one of the following formats: raising awareness or support for *BRCA*-associated cancers or *BRCA* testing, information provision on a dedicated webpage, mentioning *BRCA* on a webpage, or in published media (e.g., an article/blog/leaflet/news item/podcast/post), outreach talk/workshop, online forum, signposting, support group, or telephone helpline. All GTPs who stated they provided *BRCA1* and *BRCA2* genetic testing to UK individuals were included.

All websites were screened for eligibility by two authors (A.K., K.S.). UKO/GTP websites excluded were as follows: not UK-based/unavailable to UK residents; inaccessible (faulty link); NHS hospital websites with information relevant only to their own service(s); scientific publications; health organisations unrelated to genetics/cancer; organisations not related to *BRCA*-associated cancers; websites without information targeted towards laypeople; or news media websites. JCO websites excluded were as followed: not UK-based; solely fundraising/grant giving; interfaith/peace organisations or primarily religion-based; focusing on medical research/childhood cancers/politics/poverty/helping disadvantaged groups/servicemen/volunteering; non-*BRCA* patient groups; focusing on the holocaust; focusing on art/history/culture/architectural preservation; and schools (primary/nursery/secondary). Websites were reviewed as below.

### 2.4. Data Extraction and Website Content Evaluation

Selected websites, were reviewed, evaluated and data extracted by seven authors independently (T.A.G., K.S., A.K., M.S., S.O., N.D, A.G., Se.G., A.T.), and subsequently reviewed by three authors together (T.A.G., C.T.F., K.S.) to ensure consistency and resolve discrepancies, between September 2022 to March 2023, using a customised REDCap database.

We developed customised questionnaires (Supplementary File: B. Questionnaire: A1, A2, A3) to assess overall website *BRCA* information provision for JCO, UKO, and GTP organisations, respectively. For UKOs/JCOs these comprised seven questions (26 items) about organisational remit, relevant general *BRCA* information, *BRCA* genetic testing and cancer risk management options, further signposting, awareness raising/educational activities, carrier/family support services, genetic testing provision/access. The GTP questionnaire comprised eight questions (28 items) about organisational remit, genetic testing services offered, test information, testing service access (eligibility/referral/counselling/booking), service components and process, service costs, *BRCA* relevant clinical information, and other hereditary cancer information.

Websites were scored across five domains for *BRCA* information provided (Table 1). These domains were selected to broadly encompass what we felt someone looking for *BRCA* information would need to be able to access information, and how that information fitted with the expectation of information that is comprehensive, fully explained, and correct. Quality was added as this is an important domain and has been previously used in assessment of written information on websites [24,25,26]. These domains included (1) accessibility (2 items: search bar presence on website, number of (≤3) clicks needed to access *BRCA* information, each scored 0/1); (2) scope (5 questions [Q2–Q6], 23 items for UKO/JCO; 7 questions [Q2–Q8], 26 items for GTP); (3) depth (6 items, each scored 0 [brief/no information] or 1 [in-depth]); (4) accuracy (6 items, each scored 0 [inaccurate] or 1 [accurate]); and (5) quality of written information (assessed by the validated 16-item DISCERN-questionnaire [27,28], adapted for *BRCA1*/*BRCA2* information provision (Supplementary File: C).

Domains 1–4 were scored as $\frac{Total\;score\;for\;all\;items}{Total\;number\;of\;items}$, with a maximum score = 1 for each domain. For quality (domain 5), overall DISCERN score ranges from 1 to 5 (1 = serious shortcomings, 5 = minimal/no shortcomings) with the quality of information provided.

To facilitate overall comparisons between websites, domain scores were converted into summary scores. The DISCERN score was divided by five and then scores of all five domains were summed-up/aggregated and divided by five.

## 3. Results

**Figure 1:**
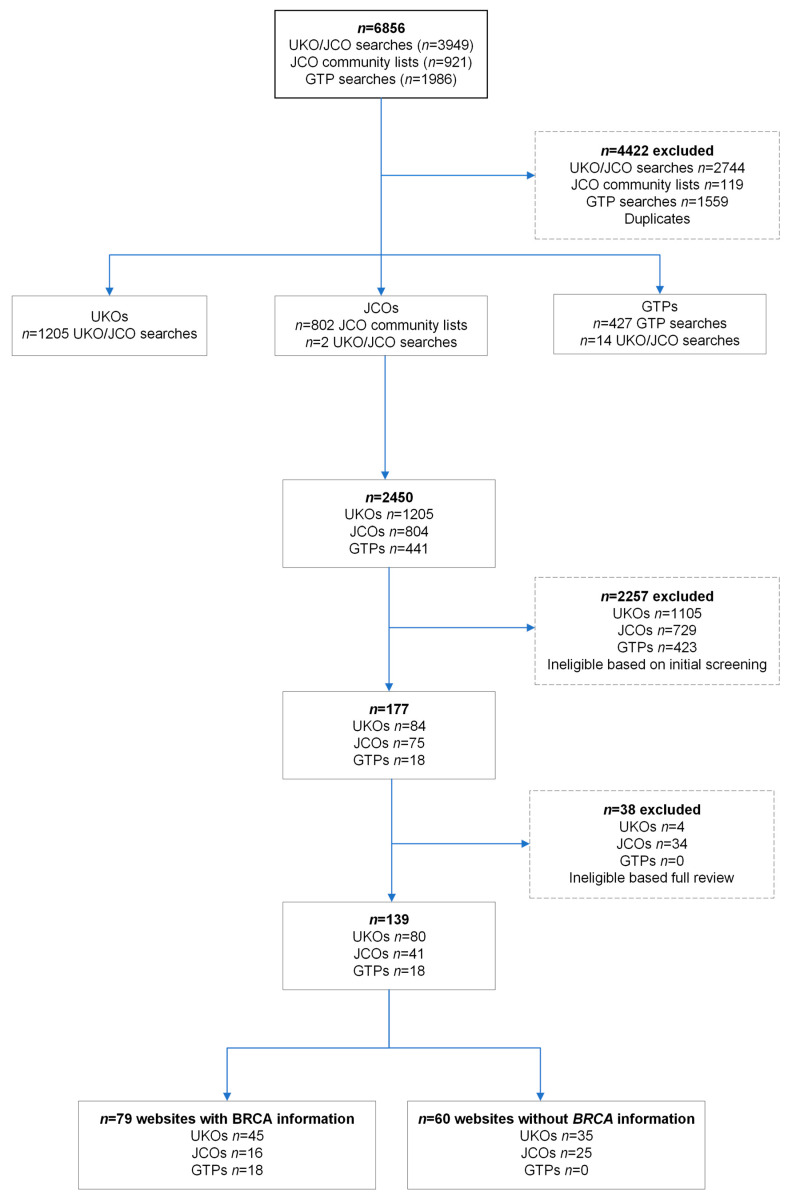
Flowchart of inclusion of UK organisations (UKOs), UK Jewish community organisations (JCOs), and genetic testing providers (GTPs).

Search results returned 6856 websites (Figure 1). Duplicates (*n* = 4422) and ineligible websites (*n* = 2257) were initially excluded. We reviewed 84 UKOs, 75 JCOs, and 18 GTPs in full, leading to 80 UKOs, 41 JCOs, and 18 GTPs as websites expected to have *BRCA* information based on their remit or organisation type (Figure 1). Of the UKOs, 73% (58/80) were organisations specific for one of the *BRCA*-associated cancers, women’s cancer, or women’s health (Supplementary Table S1). JCOs were classified as 37% (15/41) health/welfare-related, 32% (13/41) leadership, 17% (7/41) religious organisations, or 14% (6/41) educational (Supplementary Table S2). Only 45/80 (56%) UKOs and 16/41 (39%) JCOs had any information about *BRCA1/BRCA2.* Of these, 60% (27/45) of UKOs were focused on a specific *BRCA*-associated cancer or women’s health/cancer and 38% (6/16) of JCOs were health/welfare-related.

### 3.1. Information Provision: UKOs and JCOs

Of the websites, 61% (37/61) (28/45 UKOs, 9/16 JCOs) signposted to further *BRCA* resources. Table 2 lists the formats of information found on UKO and JCO websites, with mentioning of *BRCA1*, *BRCA2*, or *BRCA* in published media and signposting being the commonest.

Of UKOs, 10/45 (22%) had a dedicated *BRCA* webpage and 14/45 (31%) mentioned *BRCA1*, *BRCA2*, or *BRCA* on a webpage. Thirty-one UKOs mentioned *BRCA1*, *BRCA2*, or *BRCA* in published media and 10/31 had this as their only format of information. Six (13%) UKOs provided ≥4 formats and 13/45 (29%) provided only one format of information.

Of the JCOs, 8/16 (50%) provided information in one format, 7/16 (44%) in two formats, and only 1/16 (6%) in three different formats (Table 2). No JCOs provided an online forum or telephone helpline, only 1/16 (6%) had a support group, and only 13% (2/16) had a dedicated *BRCA* webpage.

**Table 2:** *BRCA* information provided by JCOs and UKOs.

	Organisation (Total Number)	
*BRCA* Information Format	JCO (*n* = 16)	UKO (*n* = 45)
Dedicated webpage	2 (13%)	10 (22%)
Mentioned on webpage	2 (13%)	14 (31%)
Mention in published media	8 (50%)	31 (69%)
Outreach talk or workshop	3 (19%)	4 (9%)
Online forum	0	4 (9%)
Signposting	10 (63%)	28 (62%)
Support group	1 (6%)	8 (18%)
Telephone helpline	0	6 (13%)

Percentages add up to more than 100% as organisations may provide more than one activity.

### 3.2. Genetic Testing Providers (GTPs)

Eighteen GTPs offering private *BRCA* genetic testing in the UK were included. GTPs varied widely based on type of testing provided, who could order the test, cost, pre- or post-test counselling, eligibility, and time to return results (Table 3). Clear information for these key factors was lacking across multiple websites, where it was unclear what the test included (56%, 10/18), which test to choose (28%, 5/18), and/or how to order the test (44%, 8/18). Regarding counselling, 33% (6/18) provided pre- and post-test counselling, 11% (2/18) post-test counselling only, 6% (1/18) pre-test counselling only, and 50% (9/18) lacked clarity. The test cost included counselling for 33% (6/18), did not for 28% (5/18), and lacked clarity in 39% (7/18).

### 3.3. Domain 1: Accessibility

*BRCA1*/*BRCA2* information was accessible within ≤3 clicks on 84% (66/79) of websites (37/45 UKOs, 14/16 JCOs, 15/18 GTPs; Table 4). Most (85%) websites (38/45 UKOs, 15/16 JCOs, 14/18 GTPs) had a search bar available. However, in 13% (10/79) of websites (6/45 UKOs, 3/16 JCOs, 1/18 GTPs), the *BRCA1*/*BRCA2* information within the website was not accessible through the search bar.

### 3.4. Domain 2: Scope

Overall, the scope of information available on websites was limited (Table 4). Of those with information, only 30% (18/61) of UKOs and JCOs addressed >50% (5/9) items about general *BRCA* information (Supplementary File: B, Questions 2a–i on Questionnaires A1 and A2). Of the GTPs, 56% (10/18) addressed >50% (4/7) items about *BRCA* relevant clinical information (Supplementary File: B, Questions 7a–g on Questionnaire A3).

Only 37% (29/79) of websites (31%, 14/45 UKOs; 19%, 3/16 JCOs; 67%, 12/18 GTPs) provided information covering ≥50% items (see methods) across the scope domain (Table 4). One JCO did not have information about any of the items.

**Table 4:** Summary statistics for items assessed within each of the five domains for UKOs, JCOs, and GTPs.

	Number of Websites with:	UKO		JCO		GTP		Total	
		*n* = 45	%	*n* = 16	%	*n* = 18	%	*n* = 79	%
Accessibility	≤3 mouse clicks	37	82.2	14	87.5	15	83.3	66	83.5
	A search bar present	38	84.4	15	93.8	14	77.8	67	84.8
	Accessibility score of 1	31	68.9	13	81.3	12	66.7	56	71
Scope	>50% total items addressed	14	31.1	3	18.8	12	66.7	29	36.7
	>50% total *BRCA* information	13	28.9	5	31.3	10	55.6	28	35.4
	Scope score of 1	0	0.0	0	0.0	0	0.0	0	0.0
Depth	*BRCA*-associated cancers (BC and OC, and/or ProC, PanC, or melanoma)	23	51.1	4	25.0	10	55.6	37	46.8
	All *BRCA* carrier risk management options	6	13.3	3	18.8	5	27.8	14	17.7
	Difference in carrier frequency in AJ vs non-AJ populations	0	0.0	1	6.3	0	0.0	1	1.3
	Explanation of *BRCA* inheritance	8	17.8	3	18.8	9	50.0	20	25.3
	Increased cancer risks for *BRCA* carriers for each gene and/or multiple cancers	16	35.6	1	6.3	6	33.3	23	29.1
	Signposting to >2 resources	11	24.4	4	25.0	3	16.7	18	22.8
	Depth score of 1	0	0.0	0	0.0	0	0.0	0	0.0
Accuracy	Accurate *BRCA*-associated cancers	38	84.4	8	50.0	16	88.9	62	78.5
	Accurate *BRCA* carrier risk management options	18	40.0	3	18.8	7	38.9	28	35.4
	Accurate carrier frequency in AJ population	5	11.1	5	31.3	5	27.8	15	19.0
	Accurate carrier frequency in the general population	0	0.0	3	18.8	2	11.1	5	6.3
	Accurate increased risks for *BRCA:* carriers female BC and OC	13	28.9	2	12.5	3	16.7	18	22.8
	Accurate increased risks for *BRCA:* carriers male BC, ProC, PanC, & melanoma	7	15.6	2	12.5	1	5.6	10	12.7
	Accuracy score of 1	0	0.0	1	6.3	0	0.0	1	1.3
Quality	DISCERN overall score of 5	2	4.4	0	0.0	0	0.0	2	2.5
	Quality score of 1	2	4.4	0	0.0	0	0.0	2	2.5
	Overall score >3.5 out of 5	5	11.1	2	12.5	2	11.1	9	11.4

Score of 1 means that all criteria of domain are fulfilled. See Table 1 for more information, AJ: Ashkenazi Jewish; BC: breast cancer; OC: ovarian cancer; PanC: pancreatic cancer; ProC: prostate cancer.

### 3.5. Domain 3: Depth

*BRCA1*/*BRCA2* association with BC and/or OC was mentioned in 78% (62/79) of websites (84%, 38/45 UKOs; 50%, 8/16 JCOs; 89%, 16/18 GTPs). Only 34% (27/79) of websites (51%, 23/45 UKOs; 25%, 4/16 JCOs; 56%, 10/18 GTPs) mentioned one or more of the additional associated cancers: pancreatic, prostate, and/or melanoma. *BRCA1/BRCA2/BRCA* carrier cancer risks were specified by 47% (37/79) of websites (58%, 26/45 UKOs; 19%, 3/16 JCOs; 44%, 8/18 GTPs). Regarding management options for *BRCA1*/*BRCA2* carriers, only 18% (14/79) of websites (13%, 6/45 UKOs; 19%, 3/16 JCOs; 28%, 5/18 GTPs) mentioned medical prevention, risk-reducing surgery, and screening, while 25% (20/79) mentioned only one or two of these options, and the remaining 57% lacked any information.

Inheritance pattern for *BRCA1*/*BRCA2* PVs was not explained by 75% (59/79; 82% (37/45) of UKOs; 81% (13/16) of JCOs; 50% (9/18) of GTPs. The rest either mentioned a 50% chance or autosomal dominant pattern and inheritance from either biological parent.

Signposting to further *BRCA* information was offered by 54% (43/79) of websites, but only 23% (18/79; 24%, 11/45 UKOs; 25%, 4/16 JCOs; 17%, 3/18 GTPs) signposted to >2 resources.

Overall, 76% (60/79; 80%, 36/45 UKOs; 81%, 13/16 JCOs; 61%, 11/18 GTPs) of organisations scored poorly (<0.5/1) in the depth domain.

### 3.6. Domain 4: Accuracy

The majority–82% (65/79)—of websites (87%, 39/45 UKOs; 50%, 8/16 JCOs; 89%, 16/18 GTPs) described *BRCA*-associated cancers. However, only four (5%) websites (UKOs = 3, JCO = 1) provided accurate risk estimates for all *BRCA*-associated cancers (breast, ovarian, prostate, and pancreatic cancer) by gene and sex. Overall, 42% (33/79; 49%, 22/45 UKOs; 19%, 3/16 JCOs; 44%, 8/18 GTPs) provided information about *BRCA1* and/or *BRCA2*-associated female BC and OC risks. Additionally, of these, 41% (9/22) of UKOs, 33% (1/3) of JCOs, and 63% (5/8) of GTPs provided inaccurate information (Table 1, Supplementary Table S3), with overestimation of BC risks. Only 29% (22/79) of websites (38%, 17/45 UKOs; 13%, 2/16 JCOs; 17%, 3/18 GTPs) provided information about *BRCA1*/*BRCA2*-associated male BC, prostate, pancreatic cancers, and/or melanoma risks. Of these, risk estimates were inaccurate in 59% (10/17) of UKOs, 0% (0/2) of JCOs, and 67% (2/3) of GTPs, with overestimation of male BC risks, inaccurate description of prostate cancer risks or increased sarcoma/gastric cancer risks. Melanoma risk estimates of 3–5% and 2–6% for *BRCA2* were provided by two websites. Over half (53%, 42/79) of websites (42%, 19/45 UKOs; 81%, 13/16 JCOs; 56%, 10/18 GTPs) did not have information about *BRCA*-associated cancer risks.

Only 24% (19/79) of websites reported *BRCA* carrier frequency in the general population (1:200–1:300) [30,31], of which 26% (5/19; 0%, 0/6 UKOs; 60%, 3/5 JCOs; 25%, 2/8 GTPs) were accurate. Jewish population *BRCA* carrier frequency (1:40) [32] was provided by 20% (16/79) of websites, of which 94% (15/16; 100%, 5/5 UKOs; 100%, 5/5 JCOs; 83%, 5/6 GTPs) were accurate.

Although 63% (50/79) lacked information, almost all (97%, 28/29) websites with information provided accurate information on management options for *BRCA1*/*BRCA2* PV carriers. One JCO mentioned OC screening as a management option, but this was not available/recommended under UK NHS care at that time [33,34,35].

Overall, 75% (59/79; 73%, 33/45 UKOs; 75%, 12/16 JCOs; 78%, 14/18 GTPs) of organisations scored <0.5 for accuracy.

### 3.7. Domain 5: Quality

Of *BRCA* information websites, 58% (46/79) had an overall DISCERN score = 1, indicating serious shortcomings in quality of information provision (42%, 9/45 UKOs; 75%, 12/16 JCOs; 83%, 15/18 GTPs), 11 (14%) scored = 2 (18%, 8/45 UKOs; 13%, 2/16 JCOs; 6%, 1/18 GTPs), 10 (13%) scored = 3, indicating moderate shortcomings (20%, 9/45 UKOs; 0%, 0/16 JCOs; 6%, 1/18 GTPs), and 10 (13%) scored = 4 (16%, 7/45 UKOs; 13%, 2/16 JCOs; 6%, 1/18 GTPs). Only two (2%) websites scored = 5, indicating minimal shortcomings (4%, 2/45 UKOs).

Overall, only 15% (12/79) of websites had quality information with some/minimal shortcomings.

### 3.8. Combined Scores across All Five Domains

Median scores for the five domains (accuracy, accessibility, scope, depth, and quality) across the three organisation types showed an overall low-to-moderate level of information provision (median = 1.9/5, IQR = 1.1; Table 5). Four websites (UKOs = 2; JCOs = 2) had overall scores < 1.

Five (11%) UKOs had an overall score > 3.5/5. Two each had a dedicated *BRCA* webpage or genetic testing webpages with *BRCA* information and one provided an extensive patient information leaflet.

Two (13%) JCOs had an overall score > 4/5 and were the two highest-scoring JCOs across all five domains, providing high-quality *BRCA* information. These had a dedicated webpage with *BRCA1*/*BRCA2* information, ≥2 formats of information, and one also provided a support group.

Two (11%) GTPs had an overall score > 3.5/5, and both offered pre- and post-test genetic counselling.

Overall, only 11% (9/79) of websites had combined scores > 3.5, indicating accessible, accurate, in-depth, quality information.

## 4. Discussion

### 4.1. Findings

We found that overall provision of *BRCA1*/*BRCA2* information was lacking/limited across websites that had a health- or community-facing remit and those that provided *BRCA* information.

Within the organisation types that provided information, there was a noticeable difference in the quality and quantity/breadth of information provided, varying between one brief mention of *BRCA1*/*BRCA2* and others that provide dedicated, easily accessible webpages with extensive information. Amongst GTPs, there was wide variation in testing information provided, including that relating to the nature of testing, costs, eligibility criteria, and the availability of and requirements for pre- and post-test counselling, with clear information regarding these issues lacking across multiple websites.

The overall accessibility of websites was high, but the information scope, depth, accuracy, and quality were poor for most, and only around half signposted to other information sources.

Very few websites provided complete information regarding all aspects of *BRCA* information (domain 2 [scope] and domain 3 [depth]). Inaccuracy of information was due to the information being out of date, but also not providing complete information regarding cancer risks and/or management. Only four (5%) provided accurate risk estimates for all *BRCA*-associated cancers by gene/sex and 53% lacked information about *BRCA*-associated cancer risks. Cancer risks provided should be up to date and be in line with risks provided in the UK Cancer Genetics Group guidelines [36]. Table 6 shows the risks that should be provided in public facing materials. Information on management options was lacking on 57% websites and only 18% covered both screening and medical/surgical prevention. Only 29% (22/79) of websites provided information about *BRCA1*/*BRCA2*-associated male breast, prostate, pancreatic cancers, and melanoma. Overall, 58% of websites had serious shortcomings and 27% had moderate shortcomings in the quality of information (DISCERN assessed) provided.

Combined scores from all five domains (accessibility, scope, depth, accuracy, and quality) suggested a low-to-moderate level of information provision, with large variation across websites. Only 11% of websites had a high level of information, suggesting these websites would be beneficial for individuals to access at when looking for *BRCA* information.

### 4.2. Strengths and Weaknesses

To our knowledge, this is the first study evaluating websites on the provision of *BRCA1*/*BRCA2* information, for individuals searching for health information online via a search engine. This is the first to evaluate organisational websites relating specifically to the Jewish community, as well as the general population. This is relevant, given the implementation of Jewish population-testing programmes in the UK and Israel. Validated tools to evaluate all relevant aspects of online information were unavailable, and so we developed a scoring system to facilitate a comprehensive and systematic review of website content across five domains. We considered the accessibility of information as equally important as accuracy, scope, depth, and quality for an interested individual, as this influences how likely information is to be viewed. We used the previously described ‘three click rule’ [37], which states that website information should be accessible within three clicks, based on the belief that users will stop searching if it takes them longer than this. However, some studies suggest that organisation, navigation, and structure of websites should also be taken into consideration [38,39]. We considered the number of clicks as simple and objective tool to assess this. We used a modified version of the validated DISCERN tool [27,28] to assess information quality. We attempted to minimise interpretation bias with DISCERN scoring by a senior reviewer (K.S.), training all reviewers, and review of any discordance/issues by two reviewers (T.A.G., K.S.).

We used the most common UK/worldwide search engine [40], and reviewed a large number of results, despite evidence that 91.5% of Google users stay on the first results page and only 4.8% click the second page [41] The number of relevant websites was too small to enable further statistical testing to quantitatively compare between websites. Although, we took appropriate steps to reduce biases in searches and website data extraction, we cannot guarantee results were free from bias, as search engine algorithms are not published. We restricted this analysis to websites relevant for a UK population. Further research will need to address information provision across a broader international population.

### 4.3. Interpretation

The finding that information provision varied across and within the three organisational groups is also consistent with findings in other studies on website information regarding cervical cancer, skin cancer, and BC screening [23,42,43]. If individuals have access to different information regarding a health-related topic, this can impact their decision-making process and choices they make. If information on a website is incomplete or poorly understood, it could cause concern and/or undermine this process [44].

Online information increases awareness and improves informed decision-making about genetic testing and/or risk management. Online provision of high-quality, trustworthy, relevant, and accessible information on *BRCA* is important given the large numbers of people who search for genetic testing information online. Around 44% of individuals having genetic testing for cancer susceptibility genes identified online searches as the primary source to look for additional information, outside of clinical pre- and post-test counselling, although it was difficult to identify trustworthy sources [45]. A one-size-fits-all approach to genetic testing may not be suitable, since individuals may need different amounts of information based on several different factors [46,47,48]. Hence, information provision must be tailored to individual circumstances, including desire for details.

Several studies have explored the effect of education and decision-making including uptake of genetic testing. Though few had equivocal results [44,49,50], multiple studies demonstrate information/education improves knowledge and satisfaction and reduces uncertainty and decisional conflict [14,51,52,53]. Some of these results may be impacted by the effect of in-person counselling, higher motivation, and/or pre-existing intention to test. However, this may not be representative or generalisable to those searching online, who may have a lower baseline level of information or intention to test. A US general population study found that repeated viewing of an online decision aid was associated with increased ease of decision, and increased likelihood to undergo testing [54]. Another US study of women undergoing *BRCA* genetic testing found 48% of participants decided to undergo genetic testing or not irrespective of clinician advice or attitude [46]. It has also been shown that higher *BRCA*-related knowledge increases the likelihood of accepting genetic testing [46,47]. Ensuring individuals have easy access to reliable *BRCA* information through not only clinicians but also websites can improve knowledge and decision-making regarding genetic testing.

The increasing awareness, along with expanding access and applicability of genomics and *BRCA* genetic testing to healthcare, is leading to many more individuals seeking trustworthy sources of information about testing tomake properly informed decisions. Hence, appropriate online *BRCA1*/*BRCA2* information provision is increasingly important. This is exemplified by the new NHS England Jewish population *BRCA* genetic testing programme [17], expanding testing for all women with BC diagnosis in a new London based NHS pilot [55], and falling thresholds for clinical genetic testing [56].

A number of organisations, particularly JCOs, do not provide comprehensive information about *BRCA1*/*BRCA2*. However, a high-quality website should provide clear signposting to other websites or sources of information. It is important that UKOs, especially those for cancer in general and with a specific remit for *BRCA*-associated cancers, provide a high standard of information. GTPs providing *BRCA1*/*BRCA2* genetic testing should provide comprehensive information regarding testing, especially those that offer direct-to-consumer tests. Our data highlight huge gaps in information provision that need addressing.

Although we evaluated online website information provision, this is not the only source of information people interested in obtaining *BRCA* information could use. Several studies show the use of social media for health-related information, including communication on *BRCA1* and *BRCA2* on several platforms, such as Facebook, Instagram, and Twitter [45,49,57]. Other examples of sources are telephone helplines and physical leaflets. We tried to identify these sources by capturing if this was provided by the organisations we investigated; however, an analysis of social media content was beyond the scope of this study and is a point for future research.

## 5. Conclusions

This is the first study to assess *BRCA* information provision available on websites of UK organisations (including Jewish-specific) and genetic testing providers. We find that overall provision of high-quality information is low, with large variation in quantity and quality across websites providing *BRCA* information. There is a need for improved accessible and high-quality online *BRCA* information provision, with signposting where appropriate to high-quality resources for organisations that are health- and/or community-related, and especially GTPs that offer *BRCA1*/*BRCA2* genetic testing.

## Figures and Tables

**Table 1:** Website scoring. Per domain a score between 0–1 can be awarded, up to a maximum score of 5 for all domains combined.

Domain	Description of Scoring
Accessibility	Count the amount of clicks 0—>3 mouse clicks to find *BRCA* information 1—≤3 mouse clicks to find *BRCA* information Availability of a search bar 0—No search bar 1—Search bar Score calculation Score = $\frac{Sum\;of\;the\;points\;for\;the\;amount\;of\;clicks\;and\;availibility\;of\;a\;search\;bar}{2}$
Scope	Number of questions answered/total number of questions JCOs: Questionnaire A1: questions 2–6, excluding 1 and 7. Score out of 23 UKOs: Questionnaire A2: questions 2–6, excluding 1 and 7. Score out of 23 GTPs: Questionnaire A3: questions 2–8. Score out of 26 Number of questions answered/total number of questions per section UKO and JCO: Q2: General *BRCA* info. Score out of 9 (2a–i) GTP: Q7: *BRCA* information available. Score out of 7 (7a–g) Score calculation Score = $\frac{Number\;of\;questions\;answered\;‘yes’\;on\;Questionnaire\;A1,\;A2,\;or\;A3}{Total\;number\;of\;questions\;in\;corresponding\;questionnaire}$
Depth	Included questions: *BRCA*-associated cancers 0—Brief or no information: only breast and/or ovarian 1—In depth: includes prostate, pancreas, and/or melanoma *BRCA* carrier risk management options 0—Brief or no information: 1–2 options mentioned 1—In depth: all management options (risk-reducing surgery, medication, screening) and/or other options such as lifestyle management Difference of carrier frequency between AJ and non-AJ populations 0—Brief or no information: mentions difference 1—In depth: provides estimates/numbers Explanation of *BRCA* inheritance 0—Brief or no information: Only mentions inheritance 1—In depth: Mentions 50:50/1 in 2 chance and/or dominant inheritance Increased cancer risks for *BRCA* carriers 0—Brief or no information: Only brief mention of increased risk of a *BRCA*-associated cancer or includes general stats for increased risk of a *BRCA*-associated cancer 1—In depth: Includes statistics or details that differentiate between *BRCA1* & *BRCA2* risks or includes statistics or details for different *BRCA1* & *BRCA2* risks for >2 relevant cancers Signposting to further *BRCA* information, service, and support resources 0—Brief or no information: to less than or equal to 2 resources 1—In depth: to multiple information/support resources Score calculation Score = $\frac{Number\;of\;questions\;with\;in\;depth\;answers\;on\;selected\;depth\;questions}{6}$
Accuracy *	Included questions: *BRCA*-associated cancers 0—Inaccurate: any mention of other cancers than the correct ones. 1—Accurate: needs to include breast, ovarian, melanoma, pancreatic or prostate. *BRCA* carrier risk management options 0—Inaccurate: wrong management options i.e., for example, screening for ovarian cancer 1—Accurate: appropriate chemoprevention, risk-reducing surgery or screening in line with clinical guidelines Carrier frequency/prevalence in AJ population 0—Inaccurate: Other than approximately 1:40 1—Accurate: Approximately 1:40 Carrier frequency/prevalence in general population 0—Inaccurate: anything below 1:400 1—Accurate: 1:250 or anything between 1:200—1:300 Increased cancer risks for *BRCA* carriers female breast cancer and ovarian cancer [1,2,29] 0—Inaccurate: any of the risks quoted fall outside of ranges from the literature 1—Accurate: all risks quoted fall within the ranges from the literature Increased cancer risks for *BRCA* carriers male breast cancer, prostate cancer, pancreatic cancer, and melanoma [1,2,29] 0—Inaccurate: any of the risks quoted fall outside of ranges from the literature 1—Accurate: all risks quoted fall within the ranges from the literature Score calculation Score = $\frac{Number\;of\;questions\;with\;accurate\;answers\;on\;selected\;accuracy\;questions}{5}$
Quality	DISCERN score from final question (Q16). Score calculation Score = $\frac{DISCERN\;score\;(Q16)}{5}$

* NA was given to websites without information to assess accuracy.

**Table 3:** GTP characteristics.

Organisation ID	Genes Tested	Who Orders the Test?	Cost of Test	Does the Cost Include Counselling?	Type of Counselling
GTP_160	61 gene panel (including *BRCA1* and *BRCA2*)	HCP or DTC	Unknown	Unknown	Provided by individuals doctor/clinician
GTP_161	AJ pathogenic variants and other SNPs	DTC	£149	No	Unknown
GTP_162	9 gene panel (including *BRCA1* and *BRCA2*)	DTC	£1400	Yes	Pre- and post-test
GTP_163	21, 14 or 3 gene panel (including *BRCA1*, *BRCA2*)	DTC	£490	Yes	Post-test
GTP_164	*BRCA1* and *BRCA2* only	HCP	$0 (for >75% individuals covered by insurance)	No	Unknown
GTP_165	Unknown	DTC	Unknown	Unknown	Pre-test
GTP_166	*BRCA1* and *BRCA2* or AJ PVs only	HCP	Unknown	Unknown	Unknown
GTP_167	Unknown but mentions *BRCA1* and *BRCA2*	HCP	Unknown	Unknown	Unknown
GTP_168	*BRCA1* and *BRCA2*	HCP or DTC	Unknown	Yes	Pre- and post-test
GTP_169	30 gene panel including *BRCA1* and *BRCA2*	DTC	£249	No	Unknown
GTP_170	WGS or breast panel (9 genes including *BRCA1* and *BRCA2*)	DTC for WGS	WGS £4995	Yes	Pre- and post-test
GTP_171	*BRCA1* and *BRCA2*	HCP	From £650	No	Recommended but not provided
GTP_172	*BRCA1* and *BRCA2*	DTC	£1985	Yes	Pre- and post-test
GTP_173	51 gene panel including *BRCA1* and *BRCA2*	DTC	£1300	Yes	Pre- and post-test
GTP_174	WGS	DTC	£299–£2164 + £275	No	Unknown
GTP_175	*BRCA1* and *BRCA2*	DTC	£606	Unknown	Unknown
GTP_176	30 gene panel including *BRCA1* and *BRCA2*	HCP	$258.95	Unknown	Post test
GTP_177	*BRCA1* and *BRCA2* full gene or only AJ PVs	HCP	Unknown	Unknown	Pre- and post-test

AJ: Ashkenazi Jewish; HCP: health care provider; DTC: direct-to-consumer.

**Table 5:** Combined scores across all five analysis domains.

	Domain Median (IQR)					
Organisation Type	Accessibility	Scope	Depth	Accuracy	Quality	Total
**JCO**	1 (0)	0.2 (0.3)	0 (0.2)	0.1 (0.4)	0.2 (0.05)	1.5 (0.7)
**UKO**	1 (0.5)	0.3 (0.3)	0.2 (0.2)	0.3 (0.3)	0.4 (0.4)	1.9 (1)
**GTP**	1 (0.5)	0.7 (0.3)	0.3 (0.3)	0.3 (0.2)	0.4 (0.4)	2.2 (0.9)
**Overall**	1 (0.5)	0.3 (0.4)	0.2 (0.3)	0.3 (0.2)	0.2 (0.4)	1.9 (1.1)

IQR: interquartile range.

**Table 6:** Cancer risks associated with BRCA1 and BRCA2 pathogenic variants to age 80.

	Female Cancer Risk % (95%CI)		Male Cancer Risk % (95%CI)	
Cancer Type	*BRCA1*	*BRCA2*	*BRCA1*	*BRCA2*
Breast	72% (65–79%)	69% (61–77%)	0.4% (0.1–1.5%)	4% (2–8%)
Ovarian	44% (36–53%)	17% (11–25%)	NA	NA
Prostate	NA	NA	Not elevated	27% (21–35%)
Pancreatic	Not elevated	2% (1–4%)	Not elevated	3% (2–5%)

NA: not applicable; CI: confidence interval.

## Data Availability

Additional data are available from the corresponding author on reasonable request.

## Supplementary Materials

The following supporting information can be downloaded at: https://www.mdpi.com/article/10.3390/cancers16132324/s1, Supplementary File: A. Keywords to identify websites; B. Data extraction questionnaires; C. Modified DISCERN questionnaire; Supplementary Table S1: UK organisation types; Supplementary Table S2: Jewish community organisation types; Supplementary Table S3: Scores for organisations with *BRCA*-related activities across the five categories.
