# Supplementary material for: Online Provision of BRCA1 and BRCA2 Health Information: A Search Engine Driven Systematic Web-Based Analysis
Source: Cancers (Basel). 2024 Jun 25;16(13):2324. doi: 10.3390/cancers16132324 (PMC11240379; doi:10.3390/cancers16132324)
Supplement: Supplementary file 1 [file cancers-16-02324-s001.zip › Supplementary_File.pdf]

A. Keywords to identify websites

Keywords used for web searches to identify UKO/JCO and GTP websites. The keywords were used in different combinations for a total of 80 searches as outlined in the tables. UKO searches were performed before GTP searches**.** GTP list was supplemented by GTPs identified from UKO/JCO searches.
JCO: Jewish community organisation; UKO: UK organisation; GTP: Genetic testing provider

UKO keyword searches (first 100 website links reviewed):

1. *BRCA* cancer
2. *BRCA* breast cancer
3. *BRCA* ovarian cancer
4. *BRCA* prostate cancer
5. *BRCA* pancreatic cancer
6. *BRCA* genes cancer
7. *BRCA* genes breast cancer
8. *BRCA* genes ovarian cancer
9. *BRCA* genes prostate cancer
10. *BRCA* genes pancreatic cancer
11. *BRCA* carrier cancer
12. *BRCA* carrier breast cancer
13. *BRCA* carrier ovarian cancer
14. *BRCA* carrier prostate cancer
15. *BRCA* carrier pancreatic cancer
16. *BRCA* mutations cancer
17. *BRCA* mutations breast cancer
18. *BRCA* mutations ovarian cancer
19. *BRCA* mutations prostate cancer
20. *BRCA* mutations pancreatic cancer
21. Information UK cancer
22. Information UK breast cancer
23. Information UK ovarian cancer
24. Information UK prostate cancer
25. Information UK pancreatic cancer
26. Organisation UK cancer
27. Organisation UK breast cancer
28. Organisation UK ovarian cancer
29. Organisation UK prostate cancer
30. Organisation UK pancreatic cancer
31. Charity UK cancer
32. Charity UK breast cancer
33. Charity UK ovarian cancer
34. Charity UK prostate cancer
35. Charity UK pancreatic cancer
36. Support UK cancer
37. Support UK breast cancer
38. Support UK ovarian cancer
39. Support UK prostate cancer
40. Support UK pancreatic cancer

GTP keyword searches (first 50 website links reviewed):

1. *BRCA* genetic testing
2. *BRCA* screening
3. *BRCA* gene test
4. *BRCA* test provider
5. *BRCA* testing services
6. *BRCA1 BRCA2* genetic testing
7. *BRCA1 BRCA2* screening
8. *BRCA1 BRCA2* gene test
9. *BRCA1 BRCA2* test provider
10. *BRCA1 BRCA2* testing services
11. Breast cancer genetic testing
12. Breast cancer screening
13. Breast cancer gene test
14. Breast cancer test provider
15. Breast cancer testing services
16. Ovarian cancer genetic testing
17. Ovarian cancer screening
18. Ovarian cancer gene test
19. Ovarian cancer test provider
20. Ovarian cancer testing services
21. Hereditary cancer genetic testing
22. Hereditary cancer screening
23. Hereditary cancer gene test
24. Hereditary cancer test provider
25. Hereditary cancer testing services
26. Prostate cancer genetic testing
27. Prostate cancer screening
28. Prostate cancer gene test
29. Prostate cancer test provider
30. Prostate cancer testing services
31. Pancreatic cancer genetic testing
32. Pancreatic cancer screening
33. Pancreatic cancer gene test
34. Pancreatic cancer test provider
35. Pancreatic cancer testing services
36. Cancer genetic testing
37. Cancer screening
38. Cancer gene test
39. Cancer test provider
40. Cancer testing services

B. Data extraction questionnaires

Questionnaire A1: Jewish Community Organisation (JCO) questionnaire

Researcher name		Date of website evaluation	
JCO Name		Website	

<u>Organisational Profile</u>

Category:

Leadership/Strategy /Regional Representation	Health/ Welfare	Religious Practice	Young Adult/ Professional Education	Adult Education	Other

Mission/Aims/Objectives – Organisational remit:

Core Activities – What they are, target audience, uptake/reach:

	JCO Checklist	On website Y/N
1	***Organisational Remit and Activities***	
a	*BRCA*-related activities included in JCO objectives/remit	
b	Any *BRCA*-related activities delivered by the JCO (e.g., talks, signposting, support)	
2	***General BRCA information***	
a	Basic description of a *BRCA* carrier	
b	*BRCA*-associated cancers	
c	Increased cancer risks for *BRCA* carriers	
d	*BRCA* carrier frequency in the general population	
e	*BRCA* carrier frequency in the Ashkenazi Jewish (AJ) population	
f	Carrier frequency differs in Ashkenazi Jewish (AJ) population and Jews of non-AJ origin	
g	*BRCA* carrier frequency in Jews of non-Ashkenazi origin	
h	Basic explanation or diagram of *BRCA* inheritance	
i	Additional note that risk can be passed down from father AND mother	
3	***BRCA testing and cancer risk management options***	
a	Basic description of what *BRCA* testing is	
b	Basic description of what testing involves	
c	Overview of eligibility for testing	
d	Options for *BRCA* carriers to manage their risk	
4	***Signposting***	
a	Signposting to *BRCA* testing services (NHS, private)	
b	Link to (or inclusion of) HC risk assessment tool	
c	Signposting to further *BRCA* information, service & support resources	

5	***Provision of BRCA awareness-raising or educational activities***	
a	Published *BRCA*-related information (blog, press, newsletters …)	
b	*BRCA*-related social media activities	
c	*BRCA*-related talk/s or presentation or workshops (yes/no, frequency, audience)	
d	Other	
6	***Support services for BRCA carriers / family***	
a	Counselling (genetic, general, support groups)	
b	Complimentary health (fitness, massage, reiki etc.)	
c	Practical help (advocacy, home services, funding)	
7	***Genetic testing***	
a	Offer a *BRCA* testing service (directly providing service)	

Questionnaire A2: UK Organisation (UKO) questionnaire

Researcher name		Date of website evaluation	
UKO Name		Website	

Organisational Profile

Category:

General Cancer	*BRCA*-Specific	Breast Cancer	Ovarian Cancer	Prostate Cancer	Pancreatic Cancer	Women's Cancers	Other

Mission/Aims/Objectives – Organisational remit:

Core Activities – What they are, target audience, uptake/reach:

	UKO Checklist	On websiteY/N
1	***Organisational Remit and Activities***	
a	*BRCA*-related activities included in UKO objectives/remit	
b	Any *BRCA*-related activities delivered by the UKO (e.g., talks, signposting, support)	
2	***General BRCA information***	
a	Basic description of a *BRCA* carrier	
b	*BRCA*-associated cancers	
c	Increased cancer risks for *BRCA* carriers	
d	*BRCA* carrier frequency in the general population	
e	*BRCA* carrier frequency in the Ashkenazi Jewish (AJ) population	
f	Carrier frequency differs in Ashkenazi Jewish (AJ) population and Jews of non-AJ origin	
g	*BRCA* carrier frequency in Jews of non-Ashkenazi origin	
h	Basic explanation or diagram of *BRCA* inheritance	
i	Additional note that risk can be passed down from father AND mother	
3	***BRCA testing and cancer risk management options***	
a	Basic description of what *BRCA* testing is	
b	Basic description of what testing involves	
c	Overview of eligibility for testing	
d	Options for *BRCA* carriers to manage their risk	
4	***Signposting***	
a	Signposting to *BRCA* testing services (NHS, private)	
b	Link to (or inclusion of) HC risk assessment tool	
c	Signposting to further *BRCA* information, service & support resources	
5	***Provision of BRCA awareness-raising or educational activities***	
a	Published *BRCA*-related information (blog, press, newsletters …)	

b	*BRCA*-related social media activities	
c	*BRCA*-related talk/s or presentation or workshops (yes/no, frequency, audience)	
d	Other	
6	***Support services for BRCA carriers / family***	
a	Counselling (genetic, general, support groups)	
b	Complimentary health (fitness, massage, reiki etc.)	
c	Practical help (advocacy, home services, funding)	
7	***Genetic testing***	
a	Offer a *BRCA* testing service (directly providing service)	

Questionnaire A3: Genetic testing provider (GTP) questionnaire

Researcher name		Date of website evaluation	
UKO Name		Website	

Category:	Private Testing Company (UK)	Private Testing Company (International)	Private Healthcare Service	Other

		On website Y/N
1	***About the organisation***	
a	How the GTP defines itself / mission / objectives	
2	***Genetic testing services offered***	
a	Range of genetic testing services offered	
3	***Test information - genes/mutations covered***	
**a**	Genes/mutations covered by the *BRCA* test/s being offered	
**b**	Cancers associated with mutations in the genes being tested	
**c**	Where/which lab is processing the test	
**d**	Accuracy of testing technology	
4	***BRCA testing service access***	
a	Eligibility - who is eligible for testing; any restrictions	
b	Referral – accept self-referrals or clinical referrals only (or both)	
c	Counselling – is pre and post-test counselling a pre-requisite; arranged by who	
d	Booking process – how to book a test	
e	Highlights special categories/criteria like high prevalence populations, very strong family history, etc	
5	***BRCA testing service components***	
a	Service components - clear description of what the service includes	
b	Sample collection – what is required and instructions for giving a sample	
c	Results - how the results are reported	
d	Results – when the results will be available	

e	Post-results support available	
6	***Service costs***	
a	Test costs – what is included in the price	
b	Additional service component costs	
7	***BRCA information available***	
a	*BRCA* carrier frequency – in the general population	
b	*BRCA* carrier frequency – in Ashkenazi Jews (AJ); in Jews of non-AJ origin	
c	Increased cancer risks associated with the mutations in the genes being tested	
d	Inheritance patterns – how *BRCA*-associated cancer risk is inherited	
e	*BRCA* carriers – risk of other family members having a *BRCA* mutation	
f	*BRCA* carriers – what it means to be a carrier	
g	*BRCA* carriers - options for managing risk	
h	Signposting to *BRCA* information resources and support resources for carriers	
8	***Other Jewish Hereditary Cancer related information***	
a	Basic description of and information about non-*BRCA* JHC	
b	Signposting to further HC resources	

C. Modified DISCERN questionnaire

Question No	Are the aims clear?*				
1	No		Partially		Yes
	1	2	3	4	5
	Are the **aims of the *BRCA* info content pages** set out in a clear way?<br>Look for a clear indication at the beginning of the publication of:<br>• what it is about<br>• what it is meant to cover (and what topics are meant to be excluded)<br>• who might find it useful.<br>* If the answer to Question 1 is 'No', go directly to Question 3				

Question No	Does it achieve its aims?				
2	No		Partially		Yes
	1	2	3	4	5
	Do the webpages do what they say they are aiming to do with regard to *BRCA* content?<br>HINT: Consider whether the publication provides the information it aimed to as outlined in Question 1				

Question No	Is it relevant?				
3	No		Partially		Yes
	1	2	3	4	5
	Is this website relevant in terms of addressing questions readers interested in finding out more about *BRCA* may have?<br>Consider whether:<br>• the publication addresses the questions that readers might ask<br>• recommendations and suggestions concerning treatment (*BRCA* testing) choices are realistic or appropriate.				

Question No	Is it clear what sources of information were used to compile the publication (other than the author or producer)?				
4	No		Partially		Yes
	1	2	3	4	5

<table>
<tr><td></td><td>Are reference sources/citations given for BRCA information included?

Check whether the main claims or statements made about treatment choices (BRCA info) are accompanied by a reference to the sources used as evidence, e.g. a research study or expert opinion.

• Look for a means of checking the sources used such as a bibliography/ reference list or the addresses of the experts or organisations quoted.

Rating note: In order to score a full ‘5’ the publication should fulfil both hints. Lists of additional sources of support and information (Q7) are not necessarily sources of evidence for the current publication.</td></tr>
</table>

<table>
<tr><td>Question No</td><td colspan="5">Is it clear when the information used or reported in the publication was produced?</td></tr>
<tr><td rowspan="3">5</td><td>No</td><td></td><td>Partially</td><td></td><td>Yes</td></tr>
<tr><td>1</td><td>2</td><td>3</td><td>4</td><td>5</td></tr>
<tr><td colspan="5">When was the BRCA info on the website produced? Look for:

• dates of the main sources of information used to compile the publication

• date of any revisions of the publication (but not dates of reprinting)

• date of publication (copyright date)

Rating note: The hints are placed in order of importance – in order to score a full ‘5’ the dates relating to the first hint should be found</td></tr>
</table>

<table>
<tr><td>Question No</td><td colspan="5">Is it balanced and unbiased?</td></tr>
<tr><td rowspan="3">6</td><td>No</td><td></td><td>Partially</td><td></td><td>Yes</td></tr>
<tr><td>1</td><td>2</td><td>3</td><td>4</td><td>5</td></tr>
<tr><td colspan="5">To what extent is the BRCA information content balanced? Look for:

• a clear indication of whether the publication is written from a personal or objective point of view

• evidence that a range of sources of info was used to compile the publication, e.g. more than one research study or expert

• evidence of an external assessment of the publication.

Be wary if:

• the publication focuses on the advantages or disadvantages of one particular treatment choice without reference to other possible choices

• the publication relies primarily on evidence from single cases (which may not be typical of people with this condition or of responses to a particular treatment)

• the information is presented in a sensational, emotive or alarmist way.</td></tr>
</table>

Question No	Does it (*BRCA*-info content) provide details of additional sources of support and information?				
7	No		Partially		Yes
	1	2	3	4	5
	Look for suggestions for further reading or for details of other organisations providing advice and information about the condition and treatment choices.				

Question No	Does it (*BRCA*-info content) refer to areas of uncertainty?				
8	No		Partially		Yes
	1	2	3	4	5
	Look for discussion of the gaps in knowledge or differences in expert opinion concerning treatment choices.<br>Be wary if the publication implies that a treatment choice affects everyone in the same way, e.g. 100% success rate with a particular treatment				

Rate Q9-14 interpreting 'it' as *BRCA*-info content, and 'each treatment' as *BRCA* testing (or other *BRCA*-related intervention)

The questions apply to the treatment (or treatments) described in the publication. Self-care is considered a form of treatment throughout this section.

Question No	Does it describe how each treatment works?				
9	No		Partially		Yes
	1	2	3	4	5
	Look for a description of how a treatment acts on the body to achieve its effect.				

Question No	Does it describe the benefits of each treatment?				
10	No		Partially		Yes
	1	2	3	4	5
	Benefits can include controlling or getting rid of symptoms, preventing recurrence of the condition and eliminating the condition, both short-term and long-term.				

Question No	Does it describe the risks of each treatment?				
11	No		Partially		Yes
	1	2	3	4	5
	Risks can include side-effects, complications and adverse reactions to treatment, both short-term and long-term. Accuracy/impact of taking blood?				

Question No	Does it describe what would happen if no treatment is used?				
12	No		Partially		Yes
	1	2	3	4	5
	Look for a description of the risks and benefits of postponing treatment, of watchful waiting (i.e. monitoring how the condition progresses without treatment) or of permanently forgoing treatment				

Question No	Does it describe how the treatment choices affect overall quality of life?				
13	No		Partially		Yes
	1	2	3	4	5
	Look for:<br>• description of the effects of the treatment choices on day-to-day activity<br>• description of the effects of the treatment choices on relationships with family, friends and carers.				

Question No	Is it clear that there may be more than one possible treatment choice?				
14	No		Partially		Yes
	1	2	3	4	5
	Look for:<br>• a description of who is most likely to benefit from each treatment choice mentioned, and under what circumstances<br>• suggestions of alternatives to consider or investigate further (including choices not fully described in the publication) before deciding whether to select or reject a particular treatment choice.				

Question No	Does it provide support for shared decision-making?				
15	No		Partially		Yes
	1	2	3	4	5
	Look for suggestions of things to discuss with family, friends, doctors or other health professionals concerning treatment choices.				

Overall rating of the publication

Question No	Based on the answers to all the above questions, rate the overall quality of the publication as a source of information about treatment choices				
16	Low<br><br>Serious or extensive shortcomings		Moderate<br><br>Potentially important but not serious shortcoming		High<br><br>Minimal shortcomings
	1	2	3	4	5
